# Building resilience when disaster strikes: adapting clinical trials in critical illness recovery

**DOI:** 10.1093/annalsats/aaoag028

**Published:** 2026-05-01

**Authors:** Anna G. Kalema, Felipe González-Seguel, Louisa A. Summers, Esther E. Dupont-Versteegden, Ashley A. Montgomery-Yates, Kirby P. Mayer

**Affiliations:** 1 Internal Medicine, Division of Pulmonary, Critical Care Medicine and Sleep Medicine, University of Kentucky, Lexington, KY, United States; 2 Department of Physical Therapy, University of Kentucky, Lexington, KY, United States; 3 Center for Muscle Biology, University of Kentucky, Lexington, KY, United States; 4 School of Physical Therapy, Faculty of Medicine, Universidad del Desarrollo, Santiago, Chile

## A research reality check

**Figure 1:**
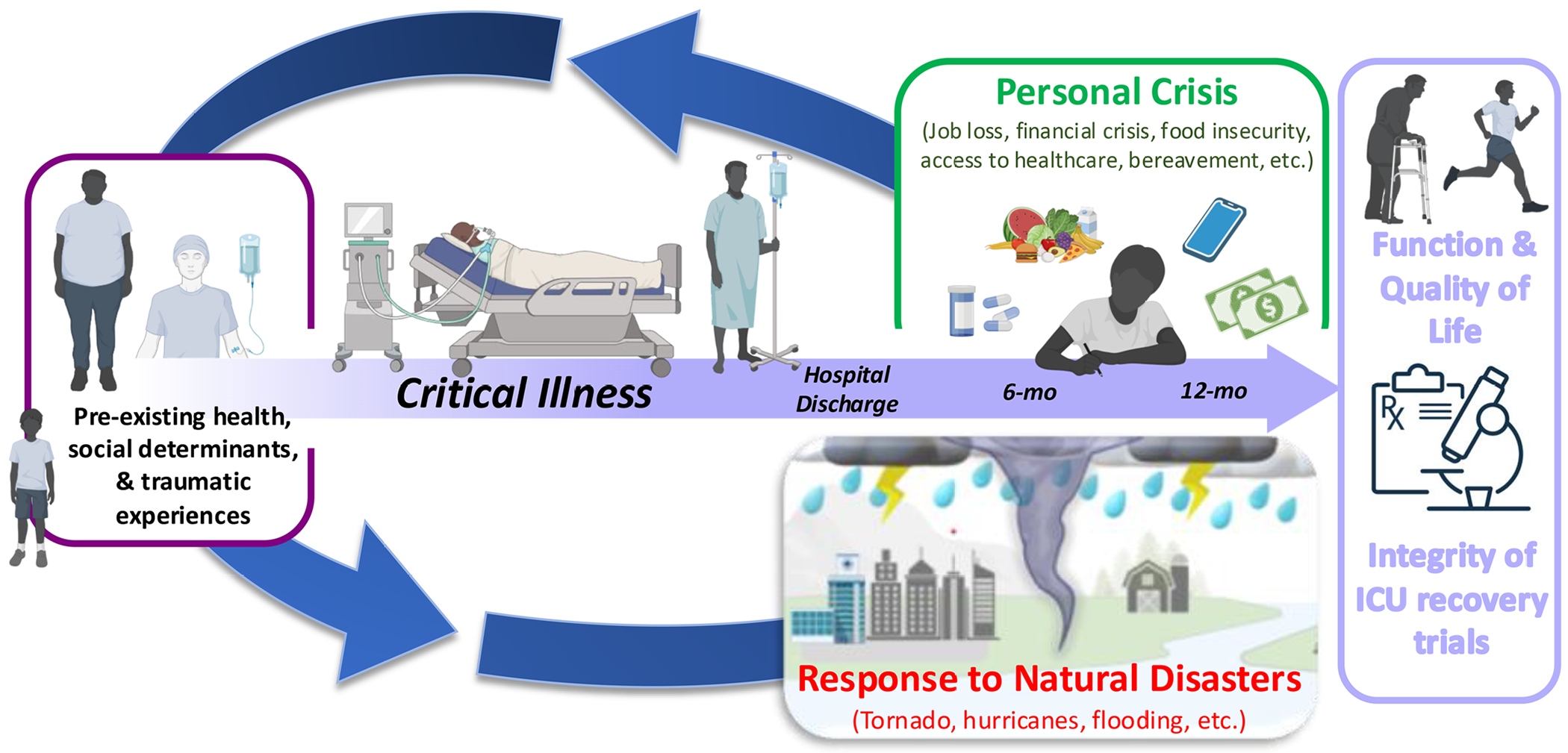
Natural disasters and personal crises influence outcomes of critical illness. The illustration is representative of how natural disasters (eg, flood, tornado, and hurricane) and personal crises (eg, loss of one’s job, food insecurity, and life stressors such as a flat tire) have the potential to confound, mediate, and modify outcomes for critical illness survivors as well as impact clinical trial integrity. The model also depicts preexisting health, and social determinants should be considered when examining long-term outcomes (eg, traumatic experience in childhood can confound, mediate, and modify outcomes). Importantly, experiences before, during, and after critical illness may influence how one responds to natural disasters and personal crises later in life. The illustration was partially created in BioRender. Dupont-Versteegden, E. (2026) https://BioRender.com/04shndz. Abbreviations: ICU, intensive care unit; mo, month.

In Kentucky, we’ve measured 6-minute walk tests in city parks, balance assessments in a fast-food restaurant, and cognitive batteries in church basements. Not because our protocol told us, but because tornadoes, floods, and hurricanes told us we had to. Studying recovery after critical illness may start in the intensive care unit (ICU), but the follow-up often unfolds in disaster zones and amid personal crises (Figure 1).

## Disasters, crisis, and their impact on trial integrity

Natural disasters and personal crises introduce far more than inconvenience.^1^ They layer trauma, disrupt health care, and destabilize research infrastructure. Events like tornadoes and flooding displace families, sever communication, and fracture the delicate chain of trial operations.^1^ For ICU recovery trials, where participants already face vulnerability from postintensive care syndrome (PICS), the effects can be especially damaging.^2^ The purpose of this provocation is to stimulate a discussion on building resilient clinical trials to address vulnerabilities that occur after hospital discharge. Before we can provide our perspective on recovery, it is imperative that we recognize that prehospital social and biologic determinants of health influence critical illness. Preexisting vulnerabilities including socioeconomic instability, life trauma, and chronic illness may influence outcomes in the recovery phase (Figure 1). Vulnerabilities throughout life may confound, mediate, and modify outcomes of PICS (Table 1). However, the focus of this provocation is on the recovery phase with ICU clinical trials frequently selecting 6 and 12 months as their primary outcome.^3^

Missed visits and selective attrition in studies with long-term outcomes are not just statistics; they carry methodological consequences.^4^ Participants most affected by crises are often those with the most impaired recovery. When these individuals drop out, studies risk underestimating the burden of long-term impairments and portraying recovery as smoother than it truly is. Endpoints such as physical performance are particularly sensitive.If the most debilitated participants vanish from the dataset, recovery trajectories become artificially optimistic.^5^

Trajectories of Recovery after Acute and Critical Illnesses (TRACER; (NCT05537298),^6^ our ongoing study of ICU survivors across Kentucky including the Appalachian region who are medically underserved and geographically isolated have lived through these realities. Within 3 years, 3 major disasters have directly disrupted our trial:

- Historic flooding (July 2022): Approximately 14-16 inches of rain fell in 5 days, killing 25 and devastating Eastern Kentucky. Two of our participants were displaced, missing critical follow-ups.
- Hurricane Helene remnants (September 2024): Though Kentucky avoided the storm’s center, regional flooding and infrastructure damage delayed multiple scheduled visits.
- Tornado classified as EF4 (Enhanced Fujita-4; ie, violent storm with wind speeds of 166-200 miles per hour, May 2025): The strongest in Kentucky since 1974 killed 12 and destroyed entire neighborhoods. Some participants became unreachable overnight, their phones disconnected as they relocated.

These events forced us into improvisation: assessments performed in restaurants, Wi–Fi borrowed from churches, or mobility testing shifted to city parks. At times, methodological adaptability and prioritization meant asking: What data matter most right now, and how do we capture them when the usual pathways collapse?

And not all crises come from the environment. More than 30% of TRACER participants experienced deep personal disruptions such as bereavement, family conflict, job loss, and even something as mundane as a flat tire. Socioeconomic hardships during and following the ICU can have a ripple effect leading to telephone services being cut off decreasing the likelihood of scheduling follow-up. While smaller in scale than a tornado, these events affected retention, performance, and outcomes. Early in TRACER, we realized that we did not have a formal protocol for capturing the concepts of resilience, optimism, or adaptability in our patients, and more importantly, we did not have a formal protocol for capturing disasters and everyday crises. Thus, we added questions on social and environmental stressors, recognizing that without them, our data would never fully reflect lived recovery.

What are the best approaches to address natural and personal disasters in studies that examine long-term outcomes? In our experience, there are multiple strategies to help control or address when disaster strikes during clinical trials. We present lessons learned from conducting TRACER with the purpose of stimulating a conversation for the critical care research community. And we welcome the opportunity for dialogue to continue to learn how we can approve our current and future research on long-term outcomes.

1. **Flexibility in the methodological approach:** Does flexibility in the methodological approach reduce the scientific rigor? Rigid follow-up windows collapse under disaster and personal crises. TRACER began a with 4-week grace period, but we quickly learned that displacement may last months. Our solution was to prioritize: if full testing was impossible, we focused on capturing primary outcomes, and less frequently we expanded the grace period to ensure data collection after the window closed. Flexibility, we believe, is not a compromise on rigor. As pragmatists at heart, we did and continue to use practical strategies to solve challenges in critical illness recovery, including strong partnership with our ICU Recovery Clinic.^7^ Importantly, we maintained our focus on in-person assessments to ensure that we can collect performance-based outcomes such as 6- minute walk tests, but we agree with the critical care field, which recognizes that telephone-based assessments provide value.^8^ As a team, we strongly believe that performance and self-reported outcomes should be aligned together to capture functional status after discharge, and thus, studies only using telecommunications may limit their ability to capture impairments. Multiple fields have demonstrated that self-reported does not always equate to performance.^9^ Thus, we utilize a hybrid approach collecting data in person and integrated phone- and telecommunication to optimize feasibility and reduce missing data. Moreover, we have plans to address missingness in our outcomes. Statistically, it is important that our field understands how missing data at random or not at random may bias outcomes impacting the trial rigor. We plan to use different approaches including adaptive imputation strategies for missing data to balance feasibility and data integrity.^10^
2. **Decentralization as methodology:** Remote and home-based assessments saved our study more than once, but they required more than good intentions. Home-based assessments require additional time and expenses for travel that quite frankly were not in the original budget. More importantly, our research team needed standardized training to ensure that a 6-minute walk test in a hotel parking lot was as valid as one in a clinic hallway. When patients were unable to travel to us, we went to them; for instance, we traveled approximately 2.5 hours to a small community in an Appalachian town of West Virigina to evaluate an individual recovering from sepsis. The person did not have internet and lived essentially in a dead zone for wireless phone connection preventing capture of data with our digital technology. Instead, we used analog methods, such as paper data collection sheets. Every adaptation to data collection or deviation to study protocol was documented in a structured format. Decentralization worked not as an emergency workaround but as a deliberate method for continuity. Finally, safety is a continuous concern when testing outside the traditional clinic or research unit. Our protocols emphasized safety in 2 domains: (1) safety of the patients and families with monitoring of vitals and symptoms with the ability to immediately stop research testing if our participants experienced distress or instability; and 2) safety of the personnel including a standard operating procedure for communicating travel to/from the testing location. Our adaptations were reported to regulatory and funding agencies, as well as notification to the Kentucky Cabinet for Healthy and Family Service for the performance of research activities in residents’ homes.
3. **Retention as relationship:** Disasters taught us that retention is not just scheduling, but rather trust. We believe participants answered our calls after a flood not because of a calendar reminder but because we had invested in relationships from day one. Communication plans included phone, text, email, and even mailed letters, but what mattered most was credibility: participants believed we would meet them where they were, even if “where” turned out to be a restaurant booth. It is important to recognize how much time is required to invest in calling (sometimes repetitively) to reach patients and their families, which is one of the big challenges for the participant retention during follow-up.^3^ Our retention strategies were developed from our experience working with leaders in our field: the Clinical Trials Network for the Prevention and Early Treatment of Acute Lung Injury (PETAL Network) with studies focusing on long-term outcomes,^11^ specifically; Dale Needham’s team who has graciously shared many lessons on maintaining rigor and quality assurance when performing long-term follow-up;^8^ Selina Parry’s team who performed falls assessments across Australia;^12^ and Peter Morris’s team who utilized an emergency medical technician^13^ to travel to participants home in their randomized controlled trial. Finally, we invested in the concept of co-design (ie, patients had a voice to help select the outcomes in our study), as the investment in the relationship with the patient and financial incentives are 2 primary staples of low retention.
4. **Research teams need resilience too:** Crisis does not spare research staff. Our team has faced illness, bereavements, and time away from work. Crises among our research team influence our ability to engage and collect patient-centered outcomes and, thus, ultimately impact trial integrity. We built redundancy by cross-training staff and enabling remote access to trial systems. This allowed continuity even when team members stepped back. Disaster resilience, we learned, is as much about protecting the workforce as protecting the participants. We recognize that we must celebrate each other’s achievements as much as we celebrate patients returning to playing with their grandchildren or going back to driving for the first time after the ICU stay.
5. **Crises is not noise:** Finally, we argue that crises should not be dismissed as background noise. Disasters and personal disruptions shape outcomes, amplify PICS symptoms, and alter recovery in ways that matter clinically. Future trials should systematically collect data on crisis exposure and incorporate it into analyses, not as an afterthought, but as a core covariate that may be a confounder, mediator, moderator, or collider factor.^14^ Failure to adapt to crises that occur in the background of clinical trials may diminish the representation of those most likely to be affected by crises.

Before we conclude, it is essential that we recognize that examining long-term outcomes in trials of critical illness is challenging even without disasters and personal crises.^15,16^

It is important, as clinicians and scientists, that we appreciate that our patients, their families, and even our research team have or will experience trauma. Adopting trauma-informed principles means creating psychologically safe environments, ensuring participant autonomy, and providing mental health referrals when distress arises. Equally, posttraumatic growth (ie, the capacity to find strength or new meaning after adversity) offers an important counterpoint to impairment-focused paradigms. We must accept that trauma has the power to confound or mediate outcomes depending on the individual.

## Conclusions: a call for a new research ethos

The future of ICU recovery trials cannot be divorced from disaster and crisis. Climate change ensures more extreme weather; socioeconomic fragility guarantees that personal crises will remain ever-present. Designing trials for an imagined “stable” world is a recipe for biased data and inequitable science.

Our experience with TRACER suggests a different path: build flexibility into protocols, embrace decentralization as methodology, treat retention as relationship, and collect crisis exposure as data. Above all, assume disruption will occur, rather than treating it as an anomaly.

If our science is to truly reflect recovery after critical illness, it must account for the messy, crisis-laden world in which recovery unfolds. Disaster-ready trials are not just practical. They are an ethical imperative for representing the vulnerable populations we aim to serve.

## Supplementary Material

Supplemental material were disclosures AMY

erial were disclosures KPM

erial were disclosures LAS

erial were disclosures AMY

erial were disclosures AGK

erial were disclosures EED

Supplementary material is available at *Annals of the American Thoracic Society* online.

## Figures and Tables

**Table 1:** Patient-related vulnerabilities influencing outcomes and trial integrity in critical illness survivorship.

Domain^a^	Descriptive	Data and outcomes^b^	Insights and opinions
**Comorbidity**	Preexisting conditions	Charlson Comorbidity Index Elix-Hauser Comorbidity Index	• Comorbid burden influences outcomes in the ICU and post-ICU phases and is necessary that trials address in their approach.
**Critical illness**	Acuity, diagnosis, treatment, and length of illness	Data collected in the ICU or extracted from electronic medical records	• The nature and severity of critical illness shape acute and long-term outcomes. Trial design should incorporate illness-related variables (SOFA, APACHE, etc.) to reduce residual confounding.
**Demographic**	Nonmodifiable patient characteristics such as age, sex, and race/ ethnicity	Data extracted from electronic medical records and/or self-reported from patient	• Differences in demographics influence care delivery and long-term outcomes. • Data extracted in medical records for clinical trials are not always accurate (ie, ethnicity listed in the medical record may not reflect how the patient identifies) and, thus, requires adjudication by patient.
**Function**	Physical, cognitive, and emotional functioning	• Proxy measures of functional status pre-ICU such as the WHODAS 2.0 • Selection of performance-based and self-reported measures in the ICU and post-ICU settings	• Highly recommend capturing or establishing prehospital functional status in long-term outcome studies. • Eligibility criteria focused on prehospital functioning may enhance internal and external validity of the sample. • Selecting outcome measures for function should be based on the scientific question and consensus recommendations. • Performance-based measures and self-report are *not* the same, and selection must consider the constructs of each measure.
**Health literacy**	Ability to obtain, process, and understand basic health information	Rapid Estimate of Adult Literacy in Medicine; Newest Vital Sign	• Health literacy affects comprehension of medical instructions and research instructions and thus trial integrity. • Low health literacy reduces engagement in care transitions and self-management, which is a critical but underrecognized resilience factor.
**Lifestyle choices**	Modifiable choices (eg, sedentary behaviors)	Subjective reports as well as proxy measures (eg, IPAQ for physical activity)	• Prehospital lifestyle may influence outcomes in the ICU and post-ICU; except for nicotine habits, lifestyle choices are not always captured in practice or clinical trials.
**Natural disasters and ecologic context**	Tornadoes, hurricanes, flooding, climate change, etc.	Self-report; data from national resources (environmental datasets)	• Disasters influence health, wellness, and outcomes. • Environmental exposures (eg, intense heat, pollution) interact with vulnerability domains and may influence post-ICU outcomes.
**Rehabilitation**	Interventions targeting recovery of function	Type, timing, and dose of rehabilitation in ICU and post-ICU settings	• Interventions in the ICU and postdischarge rehabilitation influence long-term outcomes; capturing the “dose” is critical for understanding intervention exposure and equity. Heterogeneity in the dose of rehabilitation requires research and should be considered in modeling.
**Social determinants of health**	Social, environmental, and contextual factors	Self-reported as well as tools or indices that capture social vulnerability (ADI, SVI).	• Social determinants of health influence ICU and recovery. Studies should move beyond individual-level variables to include neighborhood and system-level determinants, especially since natural disasters can be region specific (ie, hurricanes). • Disaster-related disruptions amplify disparities in social determinants of health. • Geography and access to care/research clinics lead to inequities that are frequently not addressed in trial design. • Vulnerabilities related to job loss and inability to resume driving should be captured in long-term outcomes. Financial strain post-ICU or postdisaster may prolong recovery and exacerbate inequities; economic resilience is measurable and intervenable.
**Trauma**	Traumatic events or experiences across lifespan	Self-reported or validated measures (eg, Life Events Checklist, ACE questionnaire)	• Preexisting or ICU-acquired trauma may confound, mediate, or modify recovery trajectories. Integrating trauma-informed frameworks can improve interpretation and intervention design.

a The domains are listed in alphabetical order and should be considered a representative list (not exhaustive) of the vulnerabilities that influence outcomes for ICU survivors. It is imperative to recognize that these vulnerabilities may be confounders, mediators, modifiers, or colliders to outcomes related to postintensive care syndrome as described in Figure 1 of Lederer et al.^19^

b Data and outcomes provided are examples.

Abbreviations: ICU, intensive care unit; SOFA, Sequential Organ Failure Assessment; APACHE, Acute Physiology and Chronic Health Evaluation; IPAQ, International Physical Activity Questionnaire; ADI, Area Deprivation Index; SVI, Social Vulnerability Index; ACE (questionnaire), Adverse Childhood Experiences; WHODAS, World Health Organization Disability Assessment Schedule.
